# Etiological Spectrum of Lymphadenopathy Among Children on Lymph Node Biopsy

**DOI:** 10.7759/cureus.68102

**Published:** 2024-08-29

**Authors:** Chandni Nawaz, Mulazim Hussain, Bilal Ahmad, Nighat Haider, Abdul G Khan, Muhammad Imran, Muhammad A Chaudhary

**Affiliations:** 1 Pediatric Surgery, Children’s Hospital, Pakistan Institute of Medical Sciences, Islamabad, PAK; 2 Pediatric Medicine, Children’s Hospital, Pakistan Institute of Medical Sciences, Islamabad, PAK; 3 Pediatric Medicine, Islamic International Medical College Trust, Pakistan Railway Hospital, Rawalpindi, PAK; 4 Pediatric Infectious Diseases, Children’s Hospital, Pakistan Institute of Medical Sciences, Islamabad, PAK; 5 Pediatric Medicine, Pakistan Atomic Energy Commission General Hospital, Jauharabad, PAK; 6 Pediatric Medicine, ChildLife Foundation, Islamabad, PAK

**Keywords:** pediatric, excisional biopsy, reactive hyperplasia, children, lymphoma, tuberculous lymphadenitis, lymph node, lymphadenopathy

## Abstract

Background

Pediatric peripheral lymphadenopathy is commonly a benign condition and most cases resolve spontaneously; however, it can be a manifestation of a serious underlying disease. This study aimed to determine the etiological spectrum of persistent pediatric lymphadenopathy on excisional biopsy in a tertiary care children’s hospital in a low-middle-income country and to make recommendations regarding evaluation, diagnostic testing, and surgical interventions best suited to the population.

Methodology

A prospective cross-sectional study was conducted on 243 pediatric patients between the ages of one to 12 years undergoing excisional biopsy for persistent lymphadenopathy (more than four weeks duration) from April 1, 2021, to March 31, 2024. Patient demographic data along with signs, symptoms, and results of investigations including histopathological diagnosis were documented on a structured proforma.

Results

Patients’ age range was two to 12 years (mean = 7.29 ± 2.30 years). The male-to-female ratio was 1:53. The Mean duration of lymphadenopathy was 35.89 ± 6.95 days (range = 25 to 57 days). The average size of lymph nodes ranged from 1 cm to a complex nodal mass of 7 cm. Histopathology showed reactive hyperplasia (40.32%, n = 98), tuberculosis (TB) (33.7%, n = 82), lymphoma (10.3%, n = 25), atypical mycobacterial adenitis (6.99%, n = 17), chronic granulomatous inflammation-histiocytosis (6.2%, n = 15), and Langerhans cell histiocytosis (2.5%, n = 6). The most common site was cervical. Sputum GeneXpert for TB had a true-positive rate of 78.84% while PPD was positive in only 13 TB patients. Atypical mycobacterial adenitis was successfully treated with excision and antibiotics. Supraclavicular nodes were strongly associated with lymphoma (p = 0.008).

Conclusions

Persistent pediatric lymphadenopathy is most commonly caused by TB followed by lymphoma. Positive sputum GeneXpert for TB with a suggestive clinical picture in endemic regions may be sufficient to start empiric therapy without the need for excisional biopsy in mycobacterial TB adenitis with negative PPD results and normal chest X-ray. In all other cases, excisional biopsy remains the gold standard for diagnosis. However, further studies should be conducted to formulate less invasive management algorithms.

## Introduction

Lymphadenopathy is a common presentation of multiple diseases which could be localized or generalized depending on the cause [1]. It is usually a benign condition in children as 40% of pediatric patients have palpable lymphadenopathy. The most commonly involved nodes are cervical which affect as many as 90% of children aged four to eight years [2]. Supraclavicular and inguinal involvement is less common and usually points to a grave etiology [3]. Depending on the duration of lymphadenopathy, it could be classified as acute (two weeks), subacute (two to six weeks), and chronic (more than six weeks) [3]. The etiology could be broadly classified into infectious, inflammatory, autoimmune, and malignant [3]. A good history and clinical examination are required to reach an accurate diagnosis, which includes size, site, consistency, state of the overlying skin, adherence to underlying structures, associated symptoms, exposure to animals, and duration of lymphadenopathy [2]. The cause could be evaluated further by laboratory and targeted radiological investigations. Tissue diagnosis becomes part of management when there is persistent or progressive lymphadenopathy despite antimicrobial therapy, when malignancy is suspected, and, finally, in cases of diagnostic ambiguity. Fine-needle aspiration cytology (FNAC) has an important role in adult patients with practical benefits; however, its use in children has not yet become universal due to its small sample size and the need for sedation in uncooperative patients. In addition, it carries the rare risk of needle tract seeding with cancerous cells and may have a higher false-negative rate in the diagnosis of Hodgkin’s disease [4,5]. Excisional biopsy, which is the gold standard for diagnosis, allows for the assessment of nodal architecture, providing enough tissue to perform additional investigations, including special stains, advanced flow cytometry, and karyotyping [3,6]. The drawback is that the procedure requires general anesthesia or sedation which may not be feasible in many children who are clinically unfit due to illness.

Literature states that pathologic diagnosis varies depending on the reporting group, with pediatric oncology groups reporting more malignant cases and researchers in developing countries reporting more infectious cases [4]. This study was conducted in the tertiary care Children’s Hospital, Pakistan, to evaluate the prevalence of different causes responsible for persistent lymphadenopathy on histopathology, as no such data are available locally, and to make recommendations regarding evaluation, diagnostic testing, and surgical intervention.

## Materials and methods

Study design

This prospective observational study was conducted among patients undergoing lymph node biopsy for persistent lymphadenopathy from April 2021 to March 2024 after obtaining approval from the Ethical Review Board of our institution (approval number: F.1-1/2015/ERB/SZABMU/736). The WHO sample size calculator was used to calculate a sample size of 243. Informed written consent of the parent/guardian was taken before surgery.

Inclusion and exclusion criteria

Patients aged one to 12 years coming to Children’s Hospital with lymphadenopathy requiring excisional biopsy for diagnosis were included in the study. All patients were reviewed by a pediatrician or an infectious disease specialist before referral for biopsy or were referred by the pediatric oncology team. Biopsy was performed when a significantly enlarged lymph node was noted on examination or if it progressively increased in size despite antibiotic therapy and/or when there was a clinical suspicion of tuberculosis (TB) or malignancy suggested by fixation to surrounding tissues, the persistence of lymphadenopathy beyond four weeks, presence of systemic symptoms, laboratory abnormalities, particularly anemia, leucopenia, leucocytosis, or pancytopenia, and involvement of supraclavicular node or mediastinal/hilar masses on chest X-ray. Patients with acute and subacute lymphadenitis (symptom duration of less than two to four weeks) and those undergoing lymph node sampling for established malignancy were excluded from the study.

Patients’ personal profiles along with associated symptoms and examination findings were recorded on a structured proforma. The biopsy specimens were taken into two containers, one containing normal saline for culture and sensitivity of tissue, and the other containing formalin for histopathological diagnosis.

Statistical analysis

Results were analyzed using SPSS version 26 (IBM Corp., Armonk, NY, USA). Categorical variables were expressed as frequency and percentages, while quantitative variables were calculated as mean and standard deviations. The normality of the data was analyzed using the Kolmogorov-Smirnov test. For comparison between groups, the Mann-Whitney U test and Kruskal-Wallis test were used for numerical data, and the chi-square test and Fisher’s exact test were used for categorical variables. The level of significance was set at 0.05.

## Results

The patients belonged to an age range of two to 12 years, with a mean and median of 7.29 ± 2.30 and 7.0 years, respectively. The majority (77.7%, n = 189) of the biopsies were performed between the age ranges of four to nine years. The male-to-female ratio was 1.53, with 147 males (60.5%) and 96 (39.5%) females. The mean duration of lymphadenopathy was 35.89 ± 6.95 days, with a range of 25 to 57 days. The average size of lymph nodes ranged from 1 cm to a complex nodal mass of 7 cm. More than half of the patients (57.2%, n = 139) had involvement of a single nodal group, while a large number (42.8%, n = 104) had multinodal involvement. The most common site of involvement was cervical (83.95%, n = 204), followed by inguinal (26.74%, n = 65), submandibular (25.92%, n = 63), and axillary (17.2%, n = 42) nodes. Five (2.05%) patients had supraclavicular and submental nodal involvement, while 6 (2.46%) had post-auricular lymphadenopathy.

**Figure 1:**
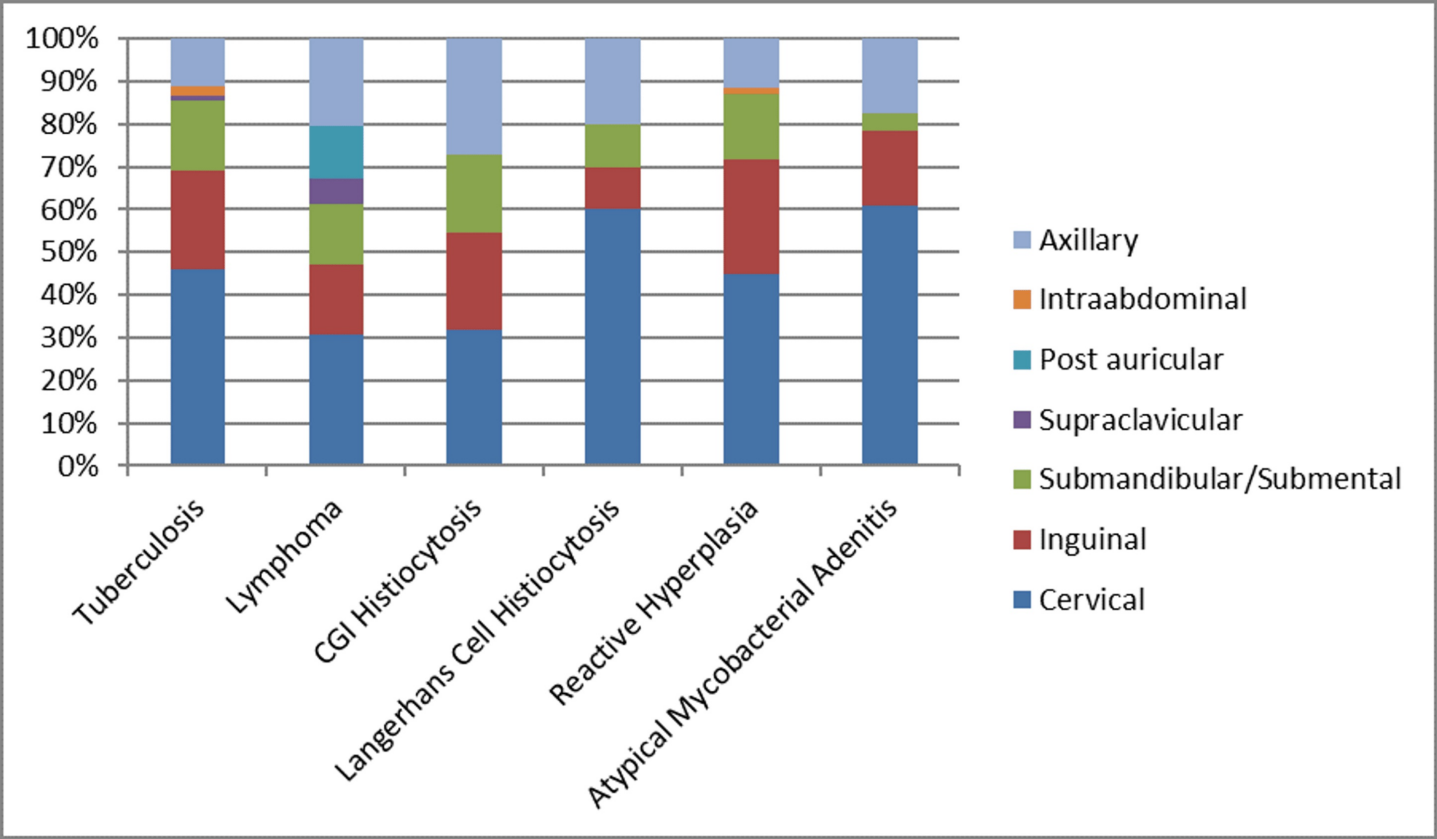
Disease-wise distribution of lymphadenopathy. CGI: chronic granulomatous inflammation

**Figure 2:**
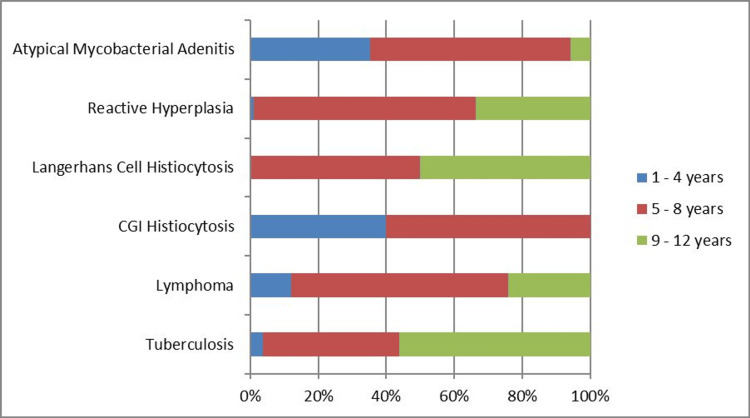
Distribution of age groups across etiologies. CGI: chronic granulomatous inflammation

A specific histopathological diagnosis was made in 145 (59.67%) patients, while the remaining biopsy specimens (40.32%, n = 98) showed reactive hyperplasia. The most common diagnosis was TB (33.7%, n = 82), followed by lymphoma (10.3%, n = 25). In total, 17 (7%) specimens demonstrated chronic granulomatous inflammation with positive acid-fast bacilli (AFB) smear but negative GeneXpert and were diagnosed as atypical mycobacterial adenitis on culture. Overall, 15 (6.2%) specimens demonstrated chronic granulomatous inflammation-histiocytosis without caseous necrosis on histopathology, while a small minority (2.5%, n = 6) showed Langerhans cell histiocytosis. Symptoms experienced by the patients are summarized in Table 1. Disease-wise distribution of lymphadenopathy is shown in Figure 1. The distribution of age across etiologies is shown in Figure 2.

**Table 1:** Association of symptoms with histopathological diagnosis Continuous variables are expressed as mean ± standard deviation. Categorical variables are expressed as frequency (n) and percentages. The level of significance is set at 0.05. *: p-value obtained by comparing groups through independent samples Kruskal-Wallis test for continuous data and the chi-square test and Fisher’s exact test for categorical data. TB: tuberculosis; RH: reactive hyperplasia; AMA: atypical mycobacterial adenitis; CGI-H: chronic granulomatous inflammation-histiocytosis; LCH: Langerhans cell histiocytosis

	TB (n = 82, 33.7%)	Lymphoma (n = 25, 10.3%)	RH (n = 98, 40.3%)	AMA (n = 17, 7%)	CGI-H (n = 15, 6.2%)	LCH (n = 6, 2.5%)	Chi-square values	P-value*
Mean age (years)	8.13 ± 2.29	6.48 ± 2.25	7.56 ± 1.81	4.76 ± 1.52	4.6 ± 2.32	8.5 ± 0.54		<0.001
Gender, male/female	42/40	19/6	66/32	8/9	6/9	6/0	12.73	0.008
Fever	75 (91.46%)	22 (88%)	21 (21.42%)	6 (35.29%)	9 (60%)	6 (100%)	106.96	<0.001
Cough	72 (87.80%)	12 (48%)	23 (23.46%)	8 (47.05%)	5 (33.33%)	6 (100%)	78.76	<0.001
Weight loss	66 (80.48%)	13 (52%)	18 (18.36%)	6 (35.29%)	5 (33.33%)	6 (100%)	76.59	<0.001
Night sweats	60 (73.17%)	12 (48%)	18 (18.36%)	6 (35.29%)	8 (53.33%)	6 (100%)	61.12	<0.001
Pallor	76 (92.68%)	24 (96%)	72 (73.46%)	16 (94.1%)	9 (60%)	6 (100%)	21.04	<0.001
Bone pains	3 (3.65%)	0	3 (3.06%)	0	0	0	1.65	1.00
Bruising	6 (7.31%)	7 (28%)	6 (6.122%)	0	5 (33.33%)	0	22.56	0.002
Positive chest findings	19 (23.17%)	15 (60%)	3 (3.06%)	2 (11.76%)	1 (6.67%)	0	51.64	<0.001

Identification of important predictors of mycobacterial tuberculosis lymphadenopathy

Positive TB contact was noted in 169 (69.5%) of 243 patients, of whom 69 (40.82%) had positive histopathological diagnosis. Three (3.6%) TB patients did not have a BCG vaccination scar. Over 90% (n = 75) of patients had systemic manifestations, and 19 (23.1%) had chest findings on physical examination and X-ray. There was a significant association (p < 0.05) between a history of TB exposure, nodal tenderness, adherence to underlying structures, matted consistency of nodes, and the presence of draining sinuses with subsequent findings of TB on histopathology. When compared using the Mann-Whitney U test, these patients had significantly higher average erythrocyte sedimentation rate (ESR) (54.92) and C-reactive protein (CRP) levels (30.46) compared to patients without TB (ESR = 44.76, CRP = 26.62, p < 0.05). The Manteaux test (TST/PPD) was performed in 131 patients (86.6% of TB patients), and it was interpreted as positive in only 13 (18.3%) of the TB patients. Before the biopsy, 71/82 (86.6%) TB patients underwent FNAC with AFB smear which was positive in 56 (78.87%) patients. Sputum GeneXpert for TB was performed in 96 (39.50%) of the 243 patients (52 TB patients); 41 (78.84% of TB) patients detected mycobacterial TB in low or very low titers, all of whom had caseous necrosis consistent with TB on histopathological analysis, while it was negative in the remaining patients.

Characteristics of patients with lymphoma on histopathology

Of the 25 patients diagnosed to have lymphoma, Hodgkin’s lymphoma was the most common subtype seen in 16 (64%) patients. Of the nine (36%) children with non-Hodgkin’s lymphoma, four patients had Burkitt lymphoma showing diffuse strong LCA, CD20 positivity, and Ki67 of 100%. One patient had diffuse large B-cell lymphoma, three patients had T-cell acute lymphoblastic lymphoma with immunohistochemistry showing Tdt (strong nuclear positive), CD3, CD10, CD20, and CD99 positivity, and Ki67 of up to 80%. One patient had peripheral T-cell lymphoma. The average duration of symptoms in these patients was 35.96 ± 6.32 days. Fifteen (60%) patients out of 25 had involvement of more than one group of nodes on presentation, while 22 (88%) patients had B symptoms. The majority of these patients had matted lymph nodes adherent to the surrounding structures (p < 0.05). This group had significantly higher CRP levels (p = 0.03).

Clinically stable patients underwent the procedure as day care surgery while the rest were kept under observation for one day following surgery. There were no complications.

## Discussion

Benign lymphadenopathy is common in the pediatric population [7]. In young children, cervical and axillary nodes may be physiologically enlarged up to a size of 1 cm, and inguinal nodes up to 1.5 cm, while supraclavicular lymph nodes are not palpable unless abnormally enlarged [4]. Pathological lymphadenopathy harkens toward an infectious, non-infectious, or malignant etiology, and the pediatric surgeon is frequently consulted to perform an excisional biopsy, particularly when there is recent-onset lymphadenopathy persisting over four to six weeks or a diagnostic dilemma. A thorough history and physical examination coupled with relevant laboratory investigations and targeted imaging is crucial in the management and must be reviewed by the surgeon before deciding on a biopsy even in properly referred cases.

In our study, the most common histopathology result was reactive adenitis which has been reported by previously published local studies as well [8]. These patients were referred back to the primary physician for further workup. It was contemplated that the chosen site of biopsy in some of these cases may not be representative of the disease process or was obscured by reactive cells [5,9]. In our institution, except in patients referred by the oncology department, the biopsy site in generalized lymphadenopathy is decided by the surgeon. We recommend multidisciplinary team meetings involving the histopathologist, oncology department, and infectious disease specialist before embarking on the surgery to ensure that proper investigations are requested and the specimen is correctly sampled, collected, and stored for processing [10].

Any substantially enlarged or progressing lymphadenopathy should always be evaluated for malignancy as part of the differential diagnosis [11]. In all such patients, a biopsy is warranted and should be performed only after a detailed medical evaluation including chest X-ray to avoid the risk of airway collapse due to compressing mediastinal masses if general anesthesia is administered [4,12]. The presence of fixed non-tender nodes, an average size of more than 3 cm, or a diffused nodal mass was significantly associated with lymphoma [13]. Literature states that palpable supraclavicular nodes are almost invariably atypical and suggestive of cancer; therefore, biopsy should be considered as soon as possible. More than half of the supraclavicular nodes in our study group demonstrated lymphoma on biopsy [11]. Moreover, it is essential to document the histopathological subtype to initiate appropriate management [14]. Hodgkin’s and non-Hodgkin’s lymphoma are the two most common causes of malignant lymphadenopathy; however, other malignancies including neuroblastoma, rhabdomyosarcoma with metastasis, malignant melanoma, and tumors of salivary glands should also be considered [3,15].

TB was the most common histopathological diagnosis. The majority of these patients reported previous exposure to a known carrier of TB and showed systemic findings of weight loss and fever, but most patients did not have chest X-ray findings of active TB. According to Spyridis et al., the presence of any two of the following three criteria leads to a diagnosis of TB lymphadenitis with 92% sensitivity: (1) a positive TST/PPD, (2) findings on chest X-ray, and (3) a history of TB contact. The PPD test was negative in a large number of our patients, which along with normal chest X-ray findings led to diagnostic uncertainty; hence, the decision to perform a biopsy was made [16]. Sputum GeneXpert for TB had a true-positive rate of 78.84% in our patients. Literature states that if performed on lymph node tissue samples, the test has positive and negative predictive values approaching 90-100% and 70-85%, respectively (specificity of 98-100%) [17]. Perhaps in the setting of available facilities in TB-endemic regions, positive sputum GeneXpert along with a suggestive clinical picture may be sufficient to start empiric antituberculous therapy with close monitoring of response, leading to avoidance of excisional biopsy altogether [17].

A proportion of our patients were diagnosed with atypical mycobacterial adenitis. Compared to the tuberculous adenitis group we noted that these were younger children (mean age = 4.76 ± 1.52 years) and all had negative PPD test results. Three of these patients had initial FNAC smear positivity for AFB and were started on antituberculous therapy. However, treatment yielded no improvement, and a biopsy was performed. Surgical excision of the lymph node mass along with antibiotic therapy resulted in successful resolution [18].

Role of fine-needle aspiration cytology

Although widely performed in adults, the apprehensive nature of a child makes it difficult to obtain an adequate sample through fine-needle aspiration (FNA). We do not prefer FNAC in our institution owing to the additional need for sedation in uncooperative children at the pathologist’s office. In our patient cohort, 167 (68.72%) had already undergone FNAC elsewhere, of which 49 smears were rendered inadequate to make a diagnosis. The true-positive rate of FNAC in diagnosing mycobacterial TB lymphadenitis was 78.87%, a number that may aid diagnosis in this group of patients when clinically correlated. However, it is not possible to differentiate between TB and atypical mycobacterial adenitis based on cytology and smear alone. Moreover performing FNA on tuberculous adenitis is controversial due to the possible risk of iatrogenic needle track fistula formation [19]. Regarding lymphoma, FNAC was performed in nine of these patients. Seven reported no atypical cells, of which four were booked for excisional biopsy while three patients were started on empiric antibiotic therapy. However, as their symptoms persisted with progression in the size of the node, an excisional biopsy was planned which confirmed malignancy. In concordance with published literature, we believe that FNAC should be altogether avoided in suspected malignant adenitis.

To summarize, excisional biopsy for pediatric lymphadenopathy should be performed at a specialized pediatric care medical institution with relevant pathology resources available to ensure that proper diagnostic studies are performed. Supraclavicular nodes, when palpable, should prompt earlier biopsy, which also holds for children with suspected malignancy [12]. We also recommend a biopsy if the lymph nodes do not regress in four weeks despite using antibiotic therapy.

Limitations

Our study has certain limitations as it is a single-center study with a sample size that is relatively small to generalize the etiological spectrum of pediatric lymphadenopathy. We had a higher percentage of malignant samples which may be because our institution is a major tertiary care referral center. Moreover, as we cater to a large number of patients from TB-endemic regions, TB lymphadenitis was the most common specific etiology identified. We acknowledge that excisional biopsy is an invasive procedure and may not be feasible in sick children due to risks associated with general anesthesia. Hence, further studies should develop and validate an algorithm to assist pediatricians in the diagnosis and timely treatment of lymphadenitis, suggesting situations where watchful waiting may be considered a safe approach, where empiric antibiotic therapy should be administered, and those requiring a timely diagnostic workup [20].

## Conclusions

Although persistent pediatric lymphadenopathy is usually a benign entity, at times, it may be the symptom of an overt or insidious disease process, with TB adenitis being the most common pathology in endemic regions, followed by lymphoma. Excisional biopsy should be considered for therapeutic intervention or as the gold standard to confirm a diagnosis or extent of the disease. However, the invasive nature of this procedure mandates the need for further studies to devise better, less invasive management algorithms.
